# Seasonality of Acute Lyme Disease in Children

**DOI:** 10.3390/tropicalmed6040196

**Published:** 2021-11-09

**Authors:** Kathryn M. Sundheim, Michael N. Levas, Fran Balamuth, Amy D. Thompson, Desiree N. Neville, Aris C. Garro, Anupam B. Kharbanda, Michael C. Monuteaux, Lise E. Nigrovic

**Affiliations:** 1 Department of Pediatrics, Boston Children’s Hospital, Boston, MA 02115, USA; 2 Division of Pediatric Emergency Medicine, Medical College of Wisconsin, Wauwatosa, WI 53226, USA; mnlevas@mcw.edu; 3 Division of Emergency Medicine, Children’s Hospital of Philadelphia, Philadelphia, PA 19104, USA; balamuthf@chop.edu; 4 Departments of Pediatrics and Emergency Medicine, Nemours/Alfred I. duPont Hospital for Children, Wilmington, DE 19899, USA; amy.thompson@nemours.org; 5 Division of Emergency Medicine, UPMC Children’s Hospital of Pittsburgh, Pittsburgh, PA 15224, USA; desiree.neville@chp.edu; 6 Departments of Pediatrics and Emergency Medicine, Rhode Island Hospital and Brown University, Providence, RI 02903, USA; arisgarro1@gmail.com; 7 Division of Emergency Medicine, Children’s Minnesota, Minneapolis, MN 55404, USA; anupam.kharbanda@childrensmn.org; 8 Division of Emergency Medicine, Boston Children’s Hospital, Boston, MA 02115, USA; michael.monuteaux@childrens.harvard.edu

**Keywords:** Lyme disease, seasonality, pediatric, arthritis

## Abstract

Due to the life cycle of its vector, Lyme disease has known seasonal variation. However, investigations focused on children have been limited. Our objective was to evaluate the seasonality of pediatric Lyme disease in three endemic regions in the United States. We enrolled children presenting to one of eight Pedi Lyme Net participating emergency departments. Cases were classified based on presenting symptoms: early (single erythema migrans (EM) lesion), early-disseminated (multiple EM lesions, headache, cranial neuropathy, or carditis), or late (arthritis). We defined a case of Lyme disease by the presence of an EM lesion or a positive two-tier Lyme disease serology. To measure seasonal variability, we estimated Fourier regression models to capture cyclical patterns in Lyme disease incidence. While most children with early or early-disseminated Lyme disease presented during the summer months, children with Lyme arthritis presented throughout the year. Clinicians should consider Lyme disease when evaluating children with acute arthritis throughout the year.

## 1. Introduction

Lyme disease is the most commonly reported vector-borne illness in the United States [1], and children are disproportionately affected [2]. In the life cycle of the *Ixodes scapularis* tick, which harbors the *Borrelia* spirochete, nymphal ticks are responsible for the majority of disease transmission and bite in the spring and the early summer. Though capable of biting throughout the year, adult ticks are much larger and thus often removed prior to disease transmission [3]. Recognizing this seasonal transmission cycle, clinicians are quick to suspect Lyme disease in the summer and early fall but may not consider Lyme disease during the winter months. However, while the symptoms of early-localized Lyme disease (single erythema migrans (EM) lesion) and early-disseminated disease (multiple EM lesions, headache, cranial neuropathy, or carditis) can present within days to weeks of a tick bite, late disease (arthritis) often manifests months later [3]. As a result, Lyme arthritis may present throughout the year, even when clinicians are less likely to consider Lyme disease. To this end, we explored the seasonality of Lyme disease overall and by clinical presentation in a multi-center cohort of children with acute Lyme disease.

## 2. Materials and Methods

We enrolled children undergoing clinical evaluation for Lyme disease presenting between 1 June 2015 and 20 July 2021 to one of the eight Pedi Lyme Net participating emergency departments, with exact enrollment dates varying by participating centers [4]. The Institutional Review Board (IRB) at each center approved the study protocol with permission for data sharing.

We defined a case of Lyme disease with either a physician-diagnosed EM lesion or a positive two-tier serology in a child with symptoms compatible with acute Lyme disease [5]. For cases of Lyme disease, patients were labeled based on the stage of presenting symptoms: early (single EM lesion), early-disseminated (multiple EM lesions, headache, cranial neuropathy, or carditis), or late (arthritis). For the purposes of this analysis, we grouped children with early and early-disseminated Lyme disease. All other children were classified as clinical mimics of Lyme disease.

We first compared children with Lyme disease to clinical mimics using the Chi square test for categorical variables and the t-test for continuous variables. Next, we compared the clinical presentations by season for children with Lyme disease versus the clinical mimics. Finally, we examined the proportion of early or early-disseminated as well as late Lyme disease cases by month of presentation. 

To measure seasonal variability in Lyme disease presentation, we estimated logistic regression models with disease status as the dependent variable and sine and cosine terms as independent variables to capture cyclical patterns in incidence (i.e., Fourier regression) [6,7]. Models were estimated separately for early or early-disseminated and late Lyme disease and were adjusted for patient age, sex, and geographic region (New England, Mid Atlantic, or Upper Midwest) with clustering by participating center. We tested the null hypothesis that the seasonal pattern for initial presentation was the same for children with early or early-disseminated versus late Lyme disease.

Analyses were performed using STATA 16.1 (StataCorp, College Station, TX, USA).

## 3. Results

Of the 3180 enrolled children, 690 (21.7%) had Lyme disease and 2490 (78.3%) were clinical mimics. Of those with Lyme disease, 77 (11.2%) had a single EM lesion, 247 (35.7%) had early-disseminated disease, and 366 (53.1%) had arthritis. Children with Lyme disease were less likely to be female but had a similar age distribution to those with alternate diagnoses (Table 1). The initial clinical presentations of children with Lyme disease differed from those of the clinical mimics.

Table 2 compares the clinical presentation by season for children with Lyme disease versus clinical mimics. Clinical mimics did not present more commonly during the peak Lyme disease season (1418/1873 (75.7%) peak season vs. 1072/1307 (82.0%) non-peak season; *p* = 0.06).

**Figure 1:**
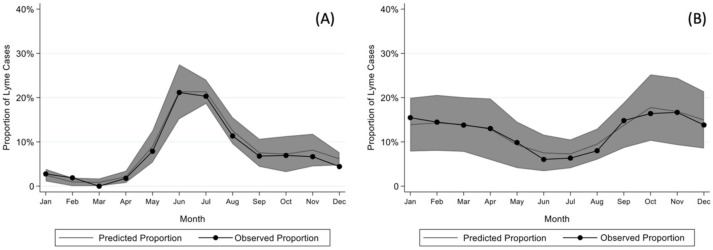
Model for seasonality of (**A**) early or early-disseminated and (**B**) late Lyme disease after adjusting for sex, age, year, and region as well as clustering by center with 95% confidence intervals.

Children with early and early-disseminated Lyme disease most frequently presented in the summer months (June through August), while children with Lyme arthritis presented throughout the year (Figure 1). The seasonality of early or early-disseminated disease significantly differed from that of late disease (*p* < 0.001).

## 4. Discussion

In our multi-center prospective study of children undergoing evaluation for acute Lyme disease, early and early-disseminated Lyme disease peaked in the summer months. However, children with Lyme arthritis presented throughout the year. For clinicians evaluating a child for potential acute Lyme disease, Lyme arthritis has to be considered throughout the whole year, while early manifestations are more likely to occur during the peak Lyme season (May to October). 

Prior work has highlighted the seasonal variation in Lyme disease presentation [2,8,9,10]; however, previously published population-based studies have been retrospective and not focused on children [2]. Pediatric studies have been small [9] or included only one disease stage [8,10,11,12]. We performed the first prospective evaluation of the seasonality of Lyme disease in children, accounting for patient level and center effects. Our next steps will be to develop tools for real-time estimate of the risk of Lyme disease based on a patient’s clinical presentation, geographic location, and date of presentation.

The most frequent Lyme disease presentation in our pediatric cohort was arthritis. In North America, Lyme disease is nearly exclusively caused by *Borrelia burgdorferi* sensu stricto, while in Europe it is predominantly caused by *Borrelia afzelii* and *Borrelia garinii*, which leads to differences in clinical manifestations [13]. Importantly, musculoskeletal manifestations of Lyme disease have been more commonly reported in North America than in Europe [14,15].

*B. burgdorferi*, the causative agent of Lyme disease, is the most recognized and reported cause of tick-borne illness; however, multiple other bacterial and parasitic organisms are also carried by the *Ioxodes scapularis* tick and are increasingly recognized as causes of human illness [16,17,18,19]. Patients with any of these tick-borne infections alone or in combination with *B. burgdorferi* can present with symptoms similar to Lyme disease [16,18,20,21]. Multiplex polymerase chain reaction panels for tick-borne coinfections have demonstrated that a substantial portion of adults with Lyme disease have coinfections [22]. However, as children are not routinely tested, the frequency of these other tick-borne infections or co-infections in children undergoing evaluation for Lyme disease is not known. Future studies are needed to understand the optimal approach to tick-borne coinfections in children.

Our study had several limitations. First, because participating centers were all located in endemic Lyme disease areas, our findings cannot be applied to non-endemic regions. Second, we enrolled patients at selected study centers when study staff were available, limiting generalizability. Third, if providers were less likely to consider Lyme disease in certain months, the seasonality we demonstrated may be partially due to providers’ decision to test rather than the true incidence of disease. Fourth, as we did not collect the duration of arthritis symptoms prior to evaluation, we cannot compare the duration of illness for children with Lyme arthritis by season. However, the vast majority of children presented to the emergency department with acute onset symptoms. Last, we may have misclassified cases of Lyme disease, as EM lesions were diagnosed by treating providers and may have included lookalike rashes (e.g., cellulitis or ring worm) and Lyme disease serology can be falsely negative early in disease or falsely positive after previous infection [23,24]. 

In conclusion, children with Lyme arthritis present throughout the year, while cases of early and early-disseminated Lyme disease peak in the summer months. Clinicians should integrate seasonal risk in the approach to children with Lyme disease. 

## Figures and Tables

**Table 1:** Comparison of clinical characteristics for children with Lyme disease versus clinical mimics.

	Lyme Disease *n* = 689 *n* (%)	Clinical Mimics *n* = 2491 *n* (%)	*p*-Value
Age (years) *	9 (4)	9 (5)	0.41
Female gender	239 (34.7)	1158 (46.5)	<0.001
*Region*			
New England	249 (36.1)	939 (37.7)	0.19
MidAtlantic	393 (56.9)	1422 (57.1)	
Upper Midwest	48 (7.0)	130 (5.2)	
*Clinical presentation*			
EM lesion	77 (11.2)	n/a	<0.001
Early-disseminated	246 (35.7)	1183 (47.5)	
Late	366 (53.1)	1198 (48.1)	
Non-specific	n/a	110 (4.4)	

* Mean (standard deviation).

**Table 2:** Comparison of children with Lyme disease and clinical mimics who presented in the peak Lyme season versus the non-peak Lyme season.

	Peak Lyme Season ^a^ *n* = 1873 *n* (%)	Non-Peak Lyme Season ^b^ *n* = 1307 *n* (%)	*p*-Value
Lyme disease (*n* = 690)			
Age (years) ^c^	9 (4)	9 (4)	0.80
Female gender	170 (34.8)	81 (34.5)	0.94
*Clinical presentation*			
EM lesion	69 (14.1)	8 (3.4)	<0.001
Early-disseminated	203 (41.5)	44 (18.7)	
Late	183 (37.4)	183 (77.9)	
Clinical mimics (*n* = 2490)			
Age (years) ^c^	9 (5)	9 (5)	0.34
*Clinical presentation*			
Early-disseminated	740 (52.2)	442 (41.2)	<0.001
Late	607 (42.8)	591 (55.2)	
Non-specific	71 (5.0)	39 (3.6)	

^a^ Presentation from 1 June to 31 October; ^b^ Presentation from 1 November to 31 May; ^c^ Mean (standard deviation).

## Data Availability

Not applicable.
